# Malaria situation in Iran: 2002–2017

**DOI:** 10.1186/s12936-019-2836-5

**Published:** 2019-06-17

**Authors:** Hassan Vatandoost, Ahmad Raeisi, Abedin Saghafipour, Fatemeh Nikpour, Jalil Nejati

**Affiliations:** 1 Department of Medical Entomology and Vector Control, School of Public Health, Tehran University of Medical Sciences, Tehran, Iran; 2 National Program for Malaria Control, Center of Disease Control & Prevention, Ministry of Health and Medical Education, Tehran, Iran; 3 Department of Public Health, School of Public Health, Qom University of Medical Sciences, Qom, Iran; 4 Health Promotion Research Center, Zahedan University of Medical Sciences, Zahedan, Iran

**Keywords:** Autochthonous, Epidemiology, Malaria, Iran

## Abstract

**Background:**

Malaria is considered as a major threat to health systems. It is still considered as one of the most important infectious diseases in Iran, but with an elimination goal in 2025. This study aimed to review the malaria situation in Iran over the 16 years.

**Methods:**

The data was collected from epidemiological registration forms that had been completed by physicians and malaria focal points in the National Centers for Disease Control and Prevention.

**Results:**

During the study period, 134,273 malaria cases were reported. The malaria incidence decreased from 0.24/1000 cases in 2002 to 0.01/1000 in 2017. From 2009 onward, the number of imported cases increased in comparison with the autochthonous and indigenous cases. Most cases were seen in males and people over 15 years of age. Moreover, the dominant registered reports were from rural areas. Most malaria cases were reported from the south and southeastern of Iran. *Plasmodium vivax* was the dominant species.

**Conclusion:**

The dramatic drop in the incidence of autochthonous cases can hopefully support malaria elimination as a major goal in the near future.

## Background

It is estimated that globally 219 million cases of malaria lead to 435,000 deaths in 2017. Most of the global malaria burden (80%) is carried by 15 countries in sub-Saharan Africa and India [1]. It is one of the most important communicable diseases transmitted by anopheline mosquitoes [2]. Currently, there are proven and effective tools to fight malaria, including vector control measures. As these tools are scaled up, malaria-endemic countries need to continually update the skills and competence of the health workers engaged in malaria control and elimination [3].

Iran is one of the malaria-endemic countries in the world, especially in the Sistan-Baluchestan and Hormozgan Provinces, in its southern and south-eastern areas, from where more than the four-fifths of cases are reported. Iran has a population of over 81 million, with a land area of 1,648,195 km^2^. Malaria is still one of the most important infectious diseases, with about 15,000 cases annually, but the total number recorded cases has dropped to less than 200, 90 and 89 locally-transmitted cases in 2015, 2016, and 2017, respectively [4–7]. The spectacular progress can be ascribed to the effective implementation of appropriate curative and preventive control interventions through a strong health care infrastructure [8]. In 2009, Iran started a malaria elimination programme with a goal to achieve this target by 2025. There has been excellent progress since, but the continued risk of importation of malaria cases from Pakistan and Afghanistan poses a huge challenge, politically, socially, operationally and technically to malaria elimination in Iran [9, 10]. The aim of this study was to analyse the malaria situation in Iran over a 16 years period.

## Methods

In this study, the data on human malaria cases were provided by the national malaria surveillance system of Iran. It included coverage of all urban and rural health centres of the country during 2002–2017. The data relevant to malaria patients including gender (male and female), nationality (Iranian, Pakistani, Afghan and other nationalities), age group (0–4, 5–14, 15 years and older) and residence place (urban, rural and nomadic population), type of human malaria parasite (*Plasmodium falciparum*, *Plasmodium vivax*, *Plasmodium malariae*, and *Plasmodium ovale*), epidemiological classification (indigenous, introduced, and relapse as autochthonous cases and also, imported and unknown) and the outcome of treatment (recovery, hospitalization, and death) were collected and analysed by SPSS version 15 (Chicago, SPSS Inc.). The mentioned terms were used based on World Health Organization (WHO) malaria terminology [11].

## Results

During the period of study, 2002–2017, 134,273 malaria cases were reported by the Iranian health system. A downward trend was seen except for some years, 2003 and 2005. In addition, malaria incidence decreased from 0.24/1000 cases in 2002 to 0.01/1000 in 2017 (Table 1).

**Table 1:** The incidence of malaria in Iran during 2002–2017

Year	Population	Cases of malaria (N)	Incidence/1000
2002	64,638,790	15,378	0.24
2003	66,480,366	25,027	0.38
2004	67,278,122	12,007	0.18
2005	68,429,945	19,285	0.28
2006	69,650,436	15,896	0.23
2007	70,924,928	16,489	0.23
2008	72,583,586	11,333	0.16
2009	73,630,366	5921	0.08
2010	73,938,131	2963	0.04
2011	75,149,669	3271	0.04
2012	76,124,600	1623	0.02
2013	76,941,000	1388	0.02
2014	77,857,000	1251	0.016
2015	78,773,000	777	0.01
2016	79,996,270	704	0.01
2017	80,000,000	960	0.012

**Fig. 1:**
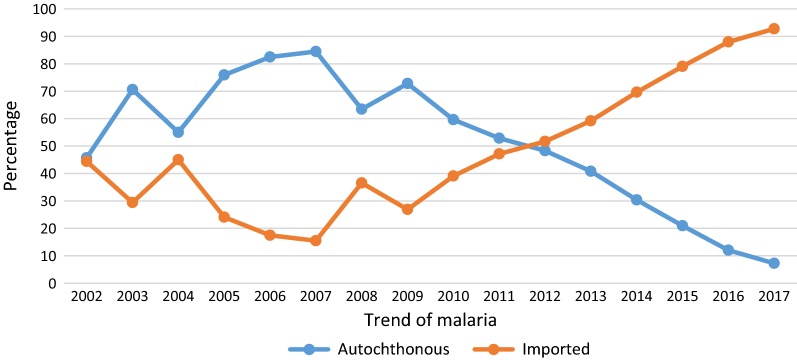
Trend of autochthonous and imported malaria cases in Iran, 2002–2017

While 18,102 autochthonous cases were reported in 2003, only 89 cases were registered in 2017 as the least number of autochthonous cases. Compared with autochthonous and indigenous cases, the number of imported cases increased from 2009 on (Table 2, Fig. 1). Most cases were observed in males and people with 15 or more years of age. Furthermore, the dominant registered reports belonged to rural areas and labourers. Most malaria cases (82.20%) were reported from three south and southeastern Iranian provinces, Sistan and Baluchestan, Hormozgan and Kerman provinces (Table 3). From a parasitical point of view, *P. vivax* was the dominant species followed by *P. falciparum*.

**Table 2:** The epidemiological classification of malaria in Iran

Year	Epidemiologic classification						
	Autochthonous^a^				Imported N (%)	Unknown N (%)	Total
	Indigenous N (%)	Introduced N (%)	Relapse N (%)	Total N (%)			
2002	6274 (40.80)	557 (3.62)	197 (1.28)	7028 (45.70)	6829 (44.41)	1521 (9.89)	15,378 (11.45)
2003	17,240 (68.88)	662 (2.65)	200 (0.80)	18,102 (72.33)	6925 (27.67)	0 (0.00)	25,027 (18.64)
2004	5245 (43.68)	332 (2.77)	206 (1.71)	5783 (48.16)	6221 (51.82)	3 (0.02)	12,007 (8.94)
2005	14,009 (72.64)	515 (2.67)	191 (0.99)	14,715 (76.30)	4570 (23.70)	0 (0.00)	19,285 (14.36)
2006	12,476 (78.48)	501 (3.16)	131 (0.82)	13,108 (82.46)	2782 (17.50)	6 (0.04)	15,896 (11.84)
2007	13,687 (83.00)	197 (1.20)	162 (0.98)	14,046 (85.18)	2434 (14.76)	9 (0.06)	16,489 (12.28)
2008	6896 (60.85)	118 (1.04)	130 (1.15)	7144 (63.04)	4189 (36.96)	0 (0.00)	11,333 (8.44)
2009	4072 (68.77)	95 (1.61)	89 (1.50)	4256 (71.88)	1645 (27.78)	20 (0.34)	5921 (4.41)
2010	1573 (53.09)	69 (2.33)	97 (3.27)	1739 (58.69)	1184 (39.96)	40 (1.35)	2963 (2.21)
2011	1612 (49.28)	78 (2.39)	54 (1.65)	1744 (53.32)	1527 (46.68)	0 (0.00)	3271 (2.44)
2012	733 (45.16)	30 (1.85)	18 (1.11)	781 (48.12)	842 (51.88)	0 (0.00)	1623 (1.21)
2013	534 (38.47)	26 (1.88)	15 (1.08)	575 (41.43)	813 (58.57)	0 (0.00)	1388 (1.03)
2014	355 (28.38)	12 (0.96)	22 (1.76)	389 (31.09)	862 (68.91)	0 (0.00)	1251 (0.93)
2015	115 (14.80)	24 (3.09)	8 (1.03)	147 (18.92)	630 (81.08)	0 (0.00)	777 (0.58)
2016	75 (10.65)	10 (1.42)	4 (0.57)	89 (12.64)	615 (87.36)	0 (0.00)	704 (0.52)
2017	69 (7.19)	17 (1.77)	3 (0.31)	89 (9.27)	871 (90.73)	0 (0.00)	960 (0.72)
Total	84,965 (63.28)	3243 (2.41)	1527 (1.14)	89,735 (66.83)	42,939 (31.98)	1599 (1.19)	134,273 (100)

^a^*Autochthonous* indigenous + introduced + relapse

**Table 3:** Demographic and clinical characteristics of malaria patients during the period of study; 2002–2017

Variables	Number (%)
Gender	
Male	88,190 (65.68)
Female	46,083 (34.32)
Age group	
0–4	9614 (7.16)
5–14	37,099 (27.63)
More than 15	87,560 (65.21)
Residency area	
Urban	40,564 (30.21)
Rural	92,165 (68.64)
Nomadic	1544 (1.15)
Nationality	
Iranian	85,344 (63.56)
Pakistan	22,209 (16.54)
Afghan	26,170 (19.49)
Others	550 (0.41)
Location of patients	
Sistan and Baluchestan, Hormozgan, Kerman	110,343 (82.20)
Other provinces	23,930 (17.82)
Occupation	
Labourer	53,937 (40.17)
Housewife	15,347 (11.43)
Student	11,172 (8.32)
Farmer	6244 (4.65)
Driver	10,232 (7.62)
Others	37,341 (27.81)
Parasite species	
*P. vivax*	116,012 (86.40)
*P. falciparum*	15,361 (11.44)
*P. malariae*	282 (0.21)
*P. vivax *+* P. falciparum*	2618 (1.95)

## Discussion

Although a high morbidity and mortality had been reported in the past decades, the recent malaria situation shows a successful elimination in most parts of the country. In 1921 and 1924, the surveys conducted by Latychev and Gilmour showed high spleen and parasite rate in the north and centre of Iran [12]. Nowadays, malaria transmission is restricted to the south and southeast of Iran, in Sistan and Baluchestan, Hormozgan and Kerman provinces, with very low incidence [13].

A malaria pre-elimination programme with WHO technical support started Iran in 2009 [14]. It was set to interrupt *P. falciparum* transmission and make Iran a malaria-free country by the end of 2015 and 2025, respectively [15]. All areas were classified into four categories based on new autochthonous malaria cases per 1000 population: (1) intensified control areas (Annual Parasite index/1000 > 5), (2) pre-elimination (5 > API/1000 > 1), (3) elimination (API/1000 < 1), (4) preventing reintroduction (without autochthonous malaria cases within 36 months). Actually, vector control and other interventions were programmed and implemented based on the class of each area [16]. “Improved access to early diagnosis and prompt treatment, expanded coverage of integrated vector management (IVM) and enhanced surveillance” were the most important strategic aspects of this programme [15]. The Iranian Ministry of Health received a Global Fund grants in 2008 and 2011 to reduce the local transmission by 80%. Despite six malaria deaths from 2012 to 2015 [17], malaria cases declined from 16,489 in 2007 to 704 in 2016. After 2017, the government contribution was the only source of malaria financing [1]. The development of a malaria early warning system, training of microscopists, rural malaria mobile teams, community volunteers, surveillance increase, operational research and building capacity of human resources have been considered as the main activities affecting this reduction [18]. Current malaria surveillance in Iran includes passive and active case finding among suspected individuals with relevant symptoms like fever. Active case finding is done by health workers (Behvarz) or volunteers. The examination of microscopic slides is used routinely for malaria diagnosis. Standard operating procedures for quality assurance of malaria diagnostic tests was performed for all positive and 10% of negative slides [19, 20]. The rapid diagnostic test (RDT), introduced in 2009, is usually used in remote areas (> 50 km from malaria diagnostic centres) among migrants, outbreaks and when microscopic detection is not possible [18]. Using this method by trained volunteers in rural communities can play an important role in early case finding [20]. Based on the national malaria treatment guidelines, chloroquine, primaquine and artesunate together with sulfadoxine/pyrimethamine are recommended as the first-line treatment for *P. vivax* and *P. falciparum*, respectively [18].

Based on the results, malaria incidence has been reduced from 333/1000 cases in 1921 to 0.01/1000 in 2017. Several studies have been conducted with the aim of finding the reasons for this decline. The improvement of socio-economic indicators, including access to electricity and water pipe network, as well as climate changes have been proposed as explanatory reasons for these changes [21]. Nevertheless, the measures taken to malaria elimination including entomological studies and interventions, such as periodic indoor residual spraying (IRS) [22, 23], insecticide resistance monitoring [24], using various larvicides [25, 26], distribution of free insecticide-treated nets (ITN) [27], and improvement tools for early detection especially using RDTs [28] can be considered as having the strongest impact on this reduction. In some studies, several factors “including increased funding, effective vector control, strengthening of health systems, improved case management with more effective treatment regimens and improved case reporting and surveillance” have been documented as the reasons of malaria elimination [29].

In the current study, malaria cases were mostly reported from three south and southeastern Iranian provinces. Most of these areas have humid and suitable weather for vectors development. Sistan and Baluchestan with an oriental climate is a vast land and has a maritime border with Pakistan in the east and Oman Sea in the south. A suitable climate for breeding various Anopheles species, including most malaria vectors, exist in this part of the country: *Anopheles stephensi*, *Anopheles culicifacies*, *Anopheles dthali*, *Anopheles fluviatilis*, *Anopheles superpictus*, *Anopheles maculipennis* and *Anopheles sacharovi* [30–33]. The first five of these vectors can be found in the southeast of the country, together with the majority of malaria cases [32]. In addition, *Anopheles pulcherrimus* has been considered as a potential malaria vector in this area based on immunological parasite detection [two-site immunoradiometric assay (IRMA)] [34]. Although all eight species may be collected in indoor places, some of them such as *An. stephensi* are considered as domestic species with endophilic behaviour. The distribution of long-lasting impregnated nets (LLINs), IRS and larviciding are applied as important strategies by Iran national malaria control programme. These free interventions are done along with other free services such as malaria diagnosis-treatment provided by the health centres [21]. Over the past years, it was proven that the high coverage of IRS application in the south of Iran has caused resistance to pyrethroid and carbamate insecticides in adult stage *An. stephensi*. Also, the resistance monitoring on the larval populations of this main malaria vector showed susceptibility to temephos [24, 35].

The results of this study showed that the imported cases increased from 2009 onward, compared to indigenous cases. Population movements, especially from the eastern neighbouring countries endemic for malaria, have been noted as an important factor. A study conducted in the south of Iran showed that the presence of foreign immigrants could cause malaria outbreaks and change the classification of cleared up and potential foci [10]. Thus, it seems that the increase in imported malaria cases is related to foreign immigrants, such as Pakistani and Afghan refugees [36]. Similarly to Iran, in Saudi Arabia, despite a decreasing trend in malaria cases between 1999 and 2010, the proportion of imported increased from 23 to 99% [29].

*Plasmodium vivax* was found to be the dominant malaria parasite species in Iran. It is reported globally and can develop in both temperate and tropical climates. Early appearance of its gametocytes, efficient transmission by Anopheles vectors at lower parasite densities, faster development of sporozoites within the mosquito and wider viable temperature ranges than *P. falciparum*, have been suggested as important reasons for the wider geographical distribution of *P. vivax*. In addition, it was documented that the vector control methods, such as ITNs were more effective in reducing *P. falciparum* transmission than *P. vivax.* In 2010, it was estimated that 2.49 billion people live in areas with the risk of *P. vivax* infection [37]. *Plasmodium vivax* is endemic in a third of the Earth’s land surface, 44 million square kilometres. Fifty-one percent of this area is located in Africa, 22% in the Americas and 27% in Asia [38]. It is believed that vivax malaria is predominant along Iran’s entire borders and in other neighbouring countries, including Afghanistan, Iraq, Oman, Pakistan, Saudi Arabia, Syria, Yemen, Armenia, Azerbaijan, Tajikistan and Turkey [39].

In the present study, most cases were seen in males and in people above 15 years of age mostly from rural areas. It has been noted that an increased proportion of malaria cases among adult men is a notable epidemiological aspect in malaria-eliminating countries. This high proportion is related to occupational and behavioural factors that cause more contact with infective vectors than women. Because of the prevalence of asymptomatic cases with low parasite densities among adult men, these so-called “hot-pops” (hot populations) can play an important role in causing outbreaks [29]. A piece of research carried out in Thailand showed that malaria can be considered as a rural disease associated with agricultural labourers [40]. A study conducted in China reported that socio-economic development is very relevant to malaria reduction. It was concluded that labourers may not have been paid enough to be able to afford malaria treatment [41]. Another study also showed that malaria in overseas Chinese labourers who returned to China represented an increasing trend in the 2001–2011 period. The researchers explained that due to global economic integration, a large number of labourers may emigrate to malaria-endemic countries [42]. However, it seems that because of free health care services, especially malaria treatment in Iran, the economic conditions of rural residents have no effect on malaria outbreaks, but their social situation can affect knowledge, attitude and practice [13].

Generally, the epidemiological classification is calculated based on a compilation of criteria [43]. The classification of malaria cases as autochthonous, indigenous, induced, imported or relapsing are very important and needs an improved surveillance health system [11]. The recognition and assessment of imported malaria cases are very important especially in the clear up foci. In addition, in malaria‐endemic areas, a misclassification of autochthonous cases instead of imported ones can affect the control programmes [44, 45]. In malaria-eliminating countries, imported malaria is as important as indigenous cases and can be considered as the main threat to achieving elimination [46]. In Italy, during 2009–2011, two autochthonous cases of malaria were considered as a threat for public health. Nevertheless, they were not proven and had been reported as probable cases [47]. These findings can show the importance of probable autochthonous cases in a non-endemic malaria setting. In fact, they resulted from an improved epidemiological survey. It seems that the improvement of the surveillance system was one of the most important activities done by the Iranian health system to eliminate malaria.

## Conclusion

The overall results of this study show that although Iran has taken great steps to eliminate malaria, it is not yet ready for certification. The dramatic drop in the incidence of autochthonous cases can be promising in supporting this major goal in the near future. Thus, high political commitment and efficient inter-sectoral collaboration are the two main factors for successful elimination.

## Data Availability

Not applicable.

## Abbreviations

ITN: insecticide-treated nets
ACT: artemisinin-based combination therapy
IRS: indoor residual spraying
RDT: rapid malaria diagnostic test
